# Percutaneous Balloon Pericardiotomy (PBP) Revisited: A Case Report and Review of Literature

**DOI:** 10.1155/2020/8121763

**Published:** 2020-05-31

**Authors:** Raed Aqel, Muawiyah Elqadi, Ahmad Hammouri, Mohammad S. Alqadi

**Affiliations:** ^1^National Center for Diabetes, Endocrinology and Genetics Center, Amman, Jordan; ^2^Al-Quds University, Faculty of Medicine, Jerusalem, State of Palestine; ^3^Al-Ahli Hospital, Hebron, State of Palestine

## Abstract

**Background:**

A Percutaneous Balloon Pericardiotomy (PBP) procedure is a reemerging nonsurgical technique that helps in preventing the reaccumulation of pericardial effusion. It is done percutaneously without general anaesthesia. It has been proved to be effective in alleviating and preventing recurrent pericardial effusion. *Case Presentation*. We reported a 52-year-old male with stage IV adenocarcinoma causing recurrent pericardial effusion. The patient experienced a worsening shortness of breath. A surgical pericardial window was denied by the surgery team secondary to severe respiratory distress; subsequently, the patient underwent Percutaneous Balloon Pericardiotomy.

**Conclusion:**

Percutaneous Balloon Pericardiotomy is efficacious and safe when done by well-trained physicians. We think it should be considered as a preferred treatment modality in most sicker patients with recurrent pericardial effusion.

## 1. Background

Recurrent large hemodynamically significant pericardial effusions, whether inflammatory or noninflammatory, remain a major health risk that can be life-threatening. Almost exclusively, these patients end in critical care units (CCU or ICU) undergoing emergency pericardial fluid aspiration mostly through catheter-based pericardiocentesis and sometimes through surgical aspiration and pericardial window creation [1].

Percutaneous Balloon Pericardiotomy (PBP) procedure is a reemerging older procedure; it is a nonsurgical technique that helps in preventing reaccumulation of pericardial effusion [1]. It is done percutaneously in the Cath suite avoiding heavy sedation and anaesthesia required for surgical window creation [2]. Initially, it was proposed by Palacios et al. in 1991 [3], after which being adopted and improved by many others [4]. Herein, we revisit this topic through a case report of PBP and review of literature.

## 2. Case Presentation

A 52-year-old male with stage IV adenocarcinoma of unknown primary, with lung and liver metastasis, presented with a worsening shortness of breath. TTE showed a large pericardial effusion pending tamponade for which he underwent emergency pericardiocentesis where 1 L of haemorrhagic fluid was drained, the drain was left in place for 48 hours then stopped, and the patient was discharged home after 48 hours. Fluid analysis showed exudative nature but no malignant cells.

Three months later, the patient presented again with a worsening shortness of breath and respiratory distress. Transthoracic echocardiogram (TTE) showed a reaccumulation of large 2.6 cm concentric pericardial effusion, with no evidence of tamponade.

A surgical pericardial window was denied by the surgery team secondary to severe respiratory distress. The patient underwent PBP. A drain was left for 24 hours after which the patient was discharged home. His subsequent clinic and TTE evaluation at 3 and 6 months revealed no more pericardial effusion accumulation.

## 3. Procedure

**Figure 1:**
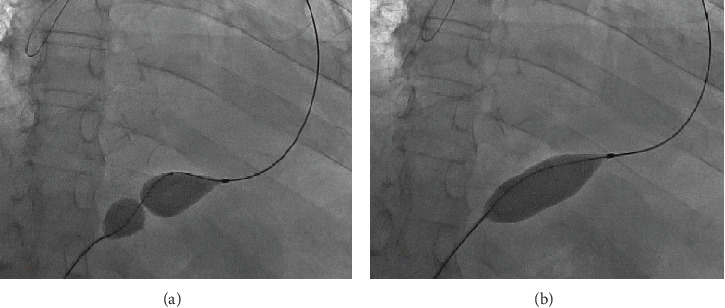
(a) Fluoroscopically showing the pericardiotomy balloon by the pericardium. (b) After balloon inflation, the balloon waist disappeared.

After administration of local anaesthesia, pericardiocentesis was performed using the classic subxiphoid approach; after aspiration of 700 cc of haemorrhagic fluid utilizing a pigtail, a stiff wire exchange was performed. Under fluoroguidance, a 2.0∗20 mm Z MED balloon, Medtronic manufacture, was placed at the pericardial edge; the balloon was inflated twice until the balloon waist disappeared (Figure 1), and a drain was left in place for 24 hours after the procedure.

Intraprocedure echocardiography showed intact cardiac champers. Follow-up CXR and TTE after 24 hours showed a mild increase in the left pleural effusion with no pericardial effusion.

## 4. Discussion

Recurrent pericardial effusion can complicate many inflammatory and noninflammatory disorders, including infectious diseases, systemic inflammatory disorders, endocrine system diseases, and chronic renal failure [5]. Moreover, disseminated malignancy can cause large pericardial effusions and can lead to cardiac tamponade [6]. Large pericardial effusions with and without tamponade is considered a clinical emergency as they might lead to progressive development of hemodynamic instability, rendering prompt diagnosis and initiation of treatment extremely essential [2].

The optimal management of pericardial effusion with tamponade remains controversial and is highly dependent on its etiology [1]. Pericardiocentesis with drainage is considered to be one of the mostly used nonsurgical treatment modalities, which can be accompanied by the installation of chemotherapeutic or sclerosing agents within the pericardial cavity [7]. Surgical approach can be used for resistant cases by the creation of a pericardial window to prevent reaccumulation [6]. The recurrence rate after pericardiocentesis is 13% to 50%, whereas it is just 4.9% after subxiphoid surgical windowing of the pericardium [7].

PBP (Percutaneous Balloon Pericardiotomy) was introduced in 1991 by Palacios et al. [3]. It provided a less invasive method than the surgical windowing with lower recurrence rates of effusion when compared with pericardiocentesis and sclerosing agents' instillation [7]. As it does not require general anaesthesia, it can be a preferred treatment modality in malignant pericardial effusions where the patients are extremely sick to undergo surgical pericardial window [8].

Despite its high success rate, which was reported to be 93% in a multicentre study of 50 patients by Ziskind et al. [7], balloon pericardiotomy has never gained a wide-spectrum acceptance because of its technical difficulty and the lack of training. Literature review revealed many case reports describing many different-etiologies large pericardial effusions treated successfully with this modality.

PBP involves the creation of a localized window or stoma between the small pericardial cavity and the spacier left pleural cavity using a percutaneous balloon-dilating catheter. The aim is to create a channel between the two cavities in an attempt to prevent the reaccumulation of fluid within the constraint pericardium [6]. This modality of treatment has proved its effectiveness in patients who present with recurrent moderate to large pericardial effusions secondary to a variety of inflammatory or noninflammatory disorders; in fact, it was even described in large recurrent pericardial effusion secondary to severe pulmonary hypertension [9]. It is generally contraindicated in infected pericardial effusions and seldomly used in this scenario [10]. This modality of treatment provides a long-term relief of pericardial effusion by shunting the fluids from the smaller pericardial space, to the bigger more forgiving pleural cavity [5].

PBP procedure is as safe as pericardiocentesis if it is done by a well-trained personnel. However, an extra caution is to be considered upon placing and inflating the pericardioplasty balloon to avoid cardiac chamber injury.

## 5. Conclusion

PBP is a safe and very effective procedure in treating and preventing the recurrence of pericardial effusion in certain groups of patients. Extra hands-on training is very essential.

## Data Availability

The datasets used in this report are available from the corresponding author on reasonable request.
